# Optimizing Staffing Level and Waiting Time Using Queuing Model in Bank Service

**DOI:** 10.1155/tswj/4133358

**Published:** 2026-05-30

**Authors:** Tadele Mamo, Dame Tolosa, S. Nagarajan, Velmurugan Paramasivam, Wosen Haile Mariam Asfaw, S. Umamaheswaran

**Affiliations:** ^1^ Department of Mechanical Engineering, Mattu University, Mattu, Ethiopia, meu.edu.et; ^2^ Faculty of Mechanical and Industrial Engineering, Bahir Dar Institute of Technology (BiT), Bahir Dar University, Bahir Dar, Ethiopia, bdu.edu.et; ^3^ Department of Artificial Intelligence and Machine Learning, New Horizon College of Engineering, Bangalore, India, newhorizonindia.edu

**Keywords:** banking service, optimization, queuing models, staffing level, waiting times

## Abstract

Long waiting times in banking services reduce customer satisfaction and operational efficiency, often resulting from misaligned staffing levels and fluctuating client traffic. The Cooperative Bank of Oromia in Holeta, Ethiopia, experiences significant service delays due to staffing inefficiencies and unpredictable customer arrivals. This study is aimed at optimizing staffing arrangements and minimize customer‐waiting times to improve service delivery. Long short‐term memory (LSTM) neural network was used to predict customer foot traffic, regression analysis assessed the impact of staffing on waiting times, and the M/M/C queuing model determined optimal staffing levels. Optimal staffing was identified as seven servers for the Holeta branch and six servers for the Goro Qeransa branch, with average system occupancy of 5.34 and 6.69 customers, respectively, leading to reduced waiting times. Aligning staffing with predicted customer flows can substantially improve service efficiency and customer satisfaction. Bank management should adopt data‐driven staffing strategies based on predictive forecasting and queuing models, which can also be adapted for similar banking contexts beyond Ethiopia.

## 1. Introduction

The evolution of global banking, as comprehensively analyzed in recent times, describes centuries of economic, political, and technological transformations [1]. Modern banking′s origins can be traced back to the Italian Renaissance, emphasizing the pivotal role of the Medici family and the significant influence of 17th‐century Dutch and English banking establishments on global financial institutions [2]. An exploration of the present‐day banking landscape emphasizes the significant changes experienced in the 21st century, driven by technological advancements and the disruptive influence of financial technology. Recent work in this area provides valuable insights into this transformative phase, highlighting the necessity for regulatory adaptations to ensure stability amid evolving financial dynamics and crises [3]. Modern banking services go beyond traditional transactions, diversifying into areas such as investment advisory, insurance, and wealth management. This aims to create a full financial ecosystem for customers [4]. Efficient banking service is vital, impacting customer satisfaction and loyalty. Seamless, timely services differentiate banks in a competitive market, fostering long‐term relationships [5]. Customer satisfaction is intricately woven into the fabric of efficient service delivery, serving as a key player for lasting loyalty [6]. Staffing dynamics in Ethiopian banking, shaped by regulatory changes and economic shifts, are consistently influenced by the National Bank of Ethiopia′s reports [7].

Research explores the application of queuing theory with in the banking industry and its broader industrial implications, emphasizing its role in optimizing service efficiency and enhancing customer satisfaction through the analysis of queue dynamics. Queuing theory finds applications in various fields, including telecommunications, traffic engineering, computer systems, manufacturing, and service industries [8]. A queuing system consists of customers arriving at a service point, forming a waiting line, and being served at a facility, with the system′s dynamics determined by the arrival and service processes [9]. Queuing theory effectively reduces customer‐waiting times at the National Bank of Iraq, leading to improved service efficiency [10]. Training benefits positively impact employee commitment and readiness for change in national banks, highlighting the need for adequate staffing to ensure optimal customer interactions and address local staff shortages [11]. Staffing challenges in banking reveal that understaffing leads to customer dissatisfaction, increased employee stress, and higher turnover, whereas over‐staffing raises labor costs and reduces productivity. The study lacks longitudinal data, limiting insights into long‐term trends and staffing effects [12].

Research on challenges in Islamic banking shows that although misdemeanor and personal issues have minimal impact on productivity, financial rewards enhance efficiency. The study does not address the long‐term effects of nonfinancial motivational factors on employee productivity [13]. Study explores the primary factors contributing to employee turnover, including job stress, satisfaction, security, work environment, and rewards. It highlights the organizational impact of turnover and emphasizes retention strategies that can improve employee motivation, productivity, and overall organizational performance [14]. The study explored how waiting line management affects service fairness and customer behavioral intention in Sharia banks, focusing on time management challenges due to rising customer transactions [15]. Virtual banks enhance services through online banking and electronic transactions, aiming to boost customer satisfaction. However, the study does not address security concerns or regulatory challenges specific to virtual banking [16]. Most clients of the Commercial Bank of Ethiopia are dissatisfied with queue management due to long waits, poor information, and uncomfortable waiting areas. However, the study does not consider variations in customer satisfaction across different branches or regions [17]. The research highlights how customer‐waiting times affect service efficiency and satisfaction in Ethiopian banks. However, it lacks an in‐depth analysis of queuing models or strategies that could optimize waiting times and improve efficiency [18]. At Cooperative Bank of Oromia, a potential gap exists between customer expectations and service delivery, requiring further study on improving service quality and satisfaction. The study lacks strategies to address this gap, limiting practical insights for improvement [19].

## 2. Materials and Method

### 2.1. Queuing Model

Queuing theory has been widely applied across different sectors, including telecommunications, traffic engineering, computer systems, manufacturing, and service industries, to analyze service processes and improve operational efficiency. In industrial production, queuing models simplify processes and support efficient system sizing to achieve quality outcomes such as zero defects [20]. In healthcare systems, queuing theory assists in identifying service bottlenecks and optimizing resource allocation to reduce waiting times and improve service delivery [21]. Correspondingly, service industries such as fast‐food outlets and public service counters use queuing models to enhance performance metrics, operational efficiency, and customer satisfaction [22, 23]. In the aerospace sector, queuing approaches are applied to optimize passenger‐handling processes, particularly at airport check‐in counters [24]. In banking services, the use of the M/M/c queuing model, which assumes Poisson arrivals and exponential service times, enables banks to estimate customer waiting times and teller utilization, supporting more effective staffing and operational planning even when detailed statistical validation is limited [25]. Queuing theory is widely applied across sectors such as telecommunications, manufacturing, healthcare, service industries, and aerospace to analyze service processes, identify bottlenecks, and improve operational efficiency and customer satisfaction. In banking, the M/M/c queuing model, based on Poisson arrivals and exponential service times, is commonly used to estimate waiting times and teller utilization, supporting effective staffing and service planning.

### 2.2. Long Short‐Term Memory Neural Network

LSTM neural networks are designed to capture long‐term dependencies in sequential data by using gated memory cells that mitigate vanishing gradients and preserve information over extended inputs [26]. In practice, LSTM models are applied by preprocessing the data, defining the network architecture, and training on historical sequences to learn complex temporal relationships [27]. Comparative studies of time‐series and deep learning models show that LSTM neural networks outperform classical ARIMA and SARIMA models in forecasting complex and nonlinear patterns. For instance, in renewable energy forecasting for Dhaka, LSTM achieved higher accuracy (R^2^ = 0.9860) and strong generalizability by effectively capturing seasonal trends and temporal dependencies, whereas ARIMA and SARIMA struggled with nonlinearity [28].

Biometric analysis of LSTM publications highlights its widespread use and growing integration with deep learning methods for enhanced predictive performance [29]. Applications span multiple domains, including modeling and forecasting future global temperature and greenhouse gas emissions [30], and predicting daily average load demand based on historical trends [31]. In healthcare, LSTM‐based approaches have been employed to determine acute ischemic stroke onset time using radiomics features from infarct lesions and whole‐brain data [32]. Hybrid models combining improved variational mode decomposition with boosting algorithms have also been proposed for passenger flow forecasting [33]. Recent advances in energy forecasting include short‐term electric load prediction using BILLET‐SimAM networks [34] and photovoltaic power prediction employing dilated causal convolution networks stacked with LSTM [35]. These examples illustrate the flexibility of LSTM neural networks in capturing temporal dependencies across diverse, nonlinear, and complex datasets.

### 2.3. Methods

The study adopts a mixed‐methods approach, combining quantitative and qualitative techniques to capture both numerical data and customer experiences. This approach integrates indicators such as customer satisfaction, staff workload, and field observations with quantitative measures including waiting times, service rates, and queue lengths. In addition, secondary information from government regulations, customer feedback records, and market reports was incorporated to support comprehensive analysis and informed decision‐making [36]. The research focuses on staff and customers of the Cooperative Bank of Oromia in Holeta town. Purposive sampling was applied to select staff members, whereas stratified random sampling was used for customers. The target population included 10–20 staff members and 400–800 customers, and the required sample size was determined using Yamane′s formula with a 95% confidence level and 5% margin of error [37]:

n=N1+Ne2,n=6001600+0.052=240

for customer k, n = sample size, N = population size, e = level of precision (5% margin of error). In this study, a confidence interval of 95% is considered, which implies that “e” is the margin of error associated with this confidence level.

### 2.4. Source and Type of Data

The study uses both primary and secondary data. Primary data is collected through direct measurements of staffing levels, waiting times, and customer feedback from interviews and surveys. This includes staffing data from office records, waiting times from queue management systems, service data from transaction logs, and customer satisfaction from surveys and observations. Secondary data comprises historical transaction data, government regulations, and existing customer feedback records.

### 2.5. Method of Data Collection

Data were collected using surveys, field observations, and semistructured interviews. Surveys gathered information on staffing levels, waiting times, and service performance, whereas field observations provided insights into customer flow and staff efficiency. Based on field observation, this study meticulously examines the average waiting time for various services, aiming to identify bottlenecks and opportunities for enhancement. Additionally, staff performance is rigorously evaluated to ensure optimal resource allocation and service quality. By applying the M/M/c queuing model to data collected from the Goro Qeransa and Holeta branches, we seek to gain valuable insights into service efficiency and staff effectiveness. The waiting time is recorded over a span of 2 weeks for each branch, with a daily sample size of 240 persons, resulting in a total observation of 240∗12 = 2880 instances for each branch. Overall, the total observations amount to 2880∗2 = 5760 raw data were collected from both branches. This comprehensive analysis, rooted in field observation, is aimed at providing actionable recommendations for optimizing service delivery and enhancing the overall customer experience in banking operations. Moreover, semistructured interviews with customers offered qualitative perspectives on service experiences, complemented by secondary data from customer feedback and market research reports. For analysis, LSTM modeling and regression techniques were applied to forecast customer demand and support resource allocation using Python and Excel. The M/M/C queuing model was employed to evaluate staffing requirements and waiting times, whereas descriptive and correlation analyses were used to assess operational performance and customer satisfaction. The reliability of multi‐item survey scales was tested using Cronbach′s alpha, which exceeded acceptable thresholds, and content validity was ensured through expert review and pilot testing to confirm that the instruments accurately measured the intended constructs [38].

### 2.6. Data Integration

**Figure 1:**
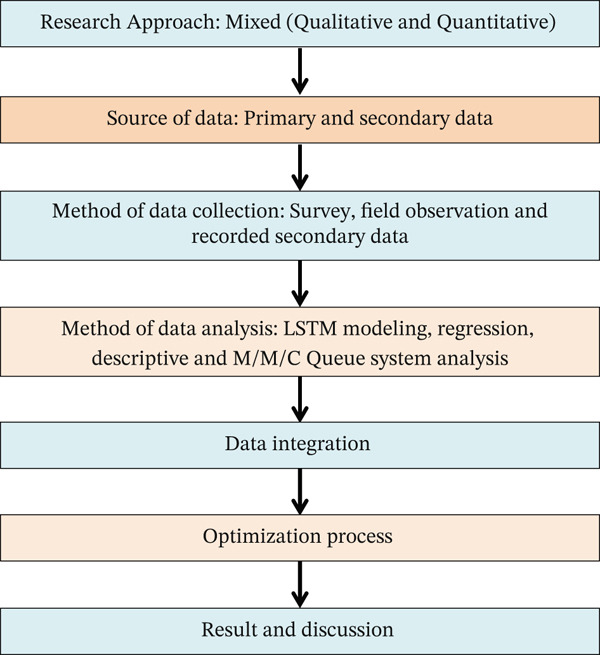
Research framework.

Combining quantitative data with qualitative insights from interviews and feedback provides a comprehensive view of staffing dynamics and service delivery. Qualitative insights from customer feedback and staff interviews were integrated with quantitative queuing metrics to provide a comprehensive interpretation of service performance; synthesizing qualitative and quantitative data is essential for enhancing the understanding of operational phenomena in survey‐based research [39]. The Research framework is shown in Figure 1.

The methodologies employed in this study encompass various advanced techniques in data modeling and optimization, facilitated by the Anaconda software environment. LSTM neural network was utilized to predict future trends in waiting times and total customer counts. Regression analysis played a crucial role in assessing the relationship between different factors and resource allocation decisions. Additionally, the M/M/C queue model was employed for queuing theory analysis, providing insights into system performance and efficiency. Throughout the analysis, fundamental libraries such as NumPy, Pandas, and Matplotlib, which are included in the Anaconda distribution, were utilized to facilitate data manipulation, analysis, and visualization. These libraries offer essential functionalities for handling diverse data‐related tasks, ensuring robustness and efficiency in the analytical process. The following detail steps were carried out in this study.

1. Data preparation is as follows:
  ■ Import pandas library as “pd” for data manipulation.
  ■ Load the monthly average customer flow data into a Data Frame.
  ■ Convert the “Month” column to datetime objects using pd.to_datetime().
  ■ Set the “Month” column as the index and sort the DataFrame by the index.
2. Data normalization is as follows:
  ■ Use MinMaxScaler from scikit‐learn to scale the “CustomerFlow” data between 0 and 1.
3. Sequence creation is as follows:
  ■ Define a function create_sequences() to generate input–output pairs from the normalized data.
  ■ Create sequences of specified length (SEQ_LENGTH) with input sequences containing the previous months′ data and output values representing the next month′s data.
4. Data splitting is as follows:
  ■ Split the sequences into training and test sets using train_test_split() from scikit‐learn.
  ■ Allocate 80% of the data for training and 20% for testing.
5. Model building is as follows:
  ■ Construct an LSTM model using the Keras Sequential API.
  ■ Design the model with two LSTM layers, each followed by a dropout layer, and a dense output layer.
  ■ Compile the model with the Adam optimizer and mean squared error (MSE) loss function.
6. Model training is as follows:
  ■ Train the LSTM model using the training data.
  ■ Specify the number of epochs, batch size, and early stopping criteria to prevent overfitting.
7. Model evaluation is as follows:
  ■ Evaluate the model′s performance on both training and test sets.
  ■ Compute MSE between actual and predicted values for evaluation.
8. Prediction is as follows:
  ■ Use the trained LSTM model to predict the next 24 months of customer flow.
  ■ Implement a function make_predictions() to iteratively predict future values based on historical data.
9. Data forecasting is as follows:
  ■ Create a DataFrame (forecast_df) containing the forecasted values for the next future months.
  ■ Index the DataFrame with datetime objects.
10. Regression analysis is as follows:
  ■ Perform exponential regression analysis to model relationships between server count, waiting times, and total customers.
  ■ Utilize linear regression on log‐transformed data for regression modeling.
11. Optimal server prediction is as follows:
  ■ Predict the optimal number of servers required to achieve a target waiting time based on regression model predictions.
  ■ Ensure the number of servers is suitable for accommodating the predicted total customers.
12. Visualization is as follows:
  ■ Visualize the results of regression analysis and optimal server prediction using Matplotib.
  ■ Plot scatter plots, regression lines, and mark the optimal server point for clarity and actionable insights.

## 3. Results and Discussion

The analysis of the Cooperative Bank of Oromia by examining survey data from staff and customers at the Goro Qeransa and Holeta branches, focusing on demographic characteristics, qualitative feedback, waiting times, and staff deployment. It utilizes diverse technological tools, including Anaconda software, Excel, and Microsoft Word, to apply the M/M/C queue system and various statistical analyses, offering actionable insights for optimizing staff and service quality.

### 3.1. Background of Cooperative Bank of Oromia S.C.

The research focuses on the Cooperative Bank of Oromia in Holeta town, examining how queuing models can optimize staffing levels and reduce customer‐waiting times to enhance service efficiency. The bank was established in 2005 to address financial exclusion in rural areas. With over 738 branches and 11.2 million account holders, the Cooperative Bank of Oromia is one of Ethiopia′s most profitable banks, addressing both historical and contemporary financial service gaps in rural communities.

### 3.2. Quantifying Relationship Between Staff Level and Waiting Time

Quantifying the link between staff levels and waiting times helps to improve service efficiency. Through statistical analysis and data‐driven strategies, businesses can optimize staffing to enhance customer satisfaction and performance. This analysis forms the basis for responsive staffing policies that address customer needs.

### 3.3. Level of Staff and Customer Satisfaction

This section analyzes staffing levels and customer satisfaction at Goro Qeransa and Holeta over 2 weeks, with 11 of 12 staff at Goro Qeransa and 16 of 19 staff at Holeta. Feedback from 210 customers helps to assess waiting times and service quality for operational improvement.

#### 3.3.1. Personal Information for Staff Survey

**Figure 2:**
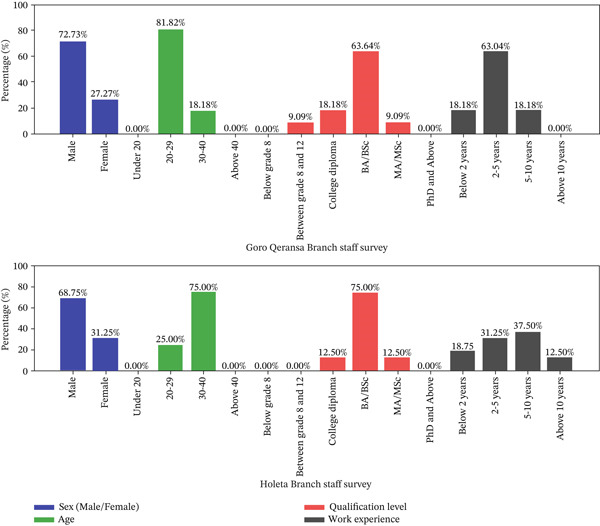
Staff demographics at Goro Qeransa and Holeta branches.

The Staff Survey Data is shown in Figure 2. It shows key demographic data as sex, age, qualifications, and experience. Goro Qeransa has more young male staff holding a BA/BSc degree. There are no employees below Grade 8, and many have limited customer service experience. Holeta has more male staff in the middle age group, with a significant number holding college diplomas and some with PhDs or higher. It also has staff members with diverse levels of experience.

#### 3.3.2. Staff Member Feedback Survey Analysis

This study analyzes 24 questions regarding resource allocation, queue length, average waiting times, and staff levels at the Goro Qeransa and Holeta branches. Section I examines resource allocation strategies, focusing on how human, financial, and technological resources are distributed to enhance efficiency and service delivery. Section II delves into queue length dynamics, exploring factors that affect customer queues, including arrival patterns and service times. Section III investigates average waiting times, identifying influences such as staffing levels and service efficiency on waiting durations. Finally, Section IV evaluates staff levels by assessing staffing adequacy through staff feedback analysis, as detailed in Table 1, which presents the results of the staff member feedback survey. Overall, staff responses indicate higher agreement than disagreement in areas such as resource allocation (51.75% agreed response for Goro Qeransa and 47.53% for Holeta branches) and average waiting times (47.16% agreed response for Goro Qeransa and 47.84% for Holeta branches), which reflects positive perceptions on resource allocation and waiting time. However, relatively higher disagreement is observed in queue length (21.86% agreed response for Goro Qeransa and 36.65% for Holeta branches) and staff levels (22.78% agreed response for Goro Qeransa and 29.58% for Holeta branches), which suggests key concern for improvement. This comprehensive approach offers valuable insights into operational performance and employee satisfaction, guiding strategic decisions to enhance service quality and efficiency in banking and similar industries.

**Table 1:** Results of the staff member feedback survey.

Staff feedback										
List of questions	Goro Qeransa branch					Holeta branch				
	Strongly disagree	Dis agree	Neutral	Agree	Strongly agree	Strongly disagree	Dis agree	Neutral	Agree	Strongly agree
Section I question related to resource allocation by organization										
Average	9.29	7.17	31.76	36.3	15.45	11.04	16.3	25.21	31	16.53
Section II question related to queue length										
Average	6.06	15.8	44.39	29.2	4.54	15.55	21.1	33.33	24.5	5.56
Section III question related to average wait times										
Average	13.13	12.7	26.97	37.8	9.36	6.67	15.6	34.45	34.5	13.34
Section IV question related to staff level										
Total average	9.48	13.3	35.5	32.9	8.8	11.58	18	33.47	27.8	9.12

#### 3.3.3. Personal Information for Customer Survey Data

**Figure 3:**
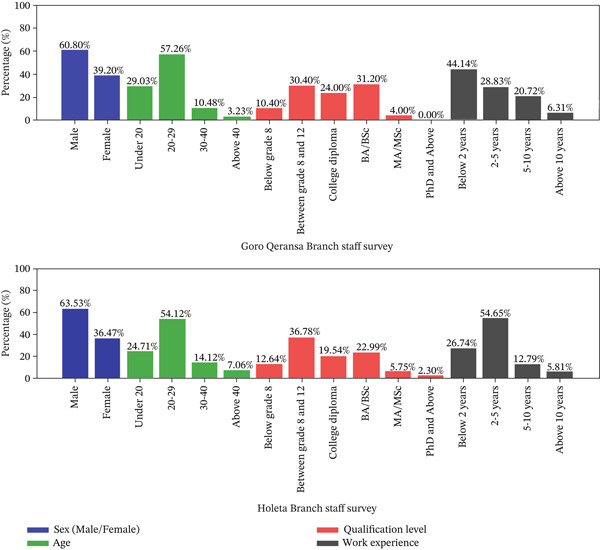
Demographic data for branch customer survey.

Analyzing customer demographics and experiences is essential for informed decision‐making. Figure 3 provides an overview of the surveyed customer base, helping businesses adapt to changing preferences and market dynamics.

#### 3.3.4. Customer Feedback Survey Analysis

The customer feedback data, outlined in Table 2, consists of 24 questions divided into four sections that focus on various aspects of service delivery. Section I assesses resource allocation by examining how effectively resources are distributed within the branches. Section II investigates customer experiences related to queue length and wait times, whereas Section III explores the average wait durations and their contributing factors. Finally, Section IV evaluates staff levels, providing insights into perceptions of staffing adequacy and effectiveness in meeting customer needs, ultimately shedding light on customer satisfaction and operational efficiency at the Goro Qeransa and Holeta branches. Therefore, customer responses show higher disagreement than agreement across most sections, particularly in resource allocation (72.74% disagreed for Goro Qeransa and 50.72% for Holeta branches) and queue length (71.43% disagreed for Goro Qeransa and 54.88% for Holeta branches), which indicates negative perceptions on resource allocation and waiting time. In total, disagreement levels (62.16% for Goro Qeransa and 50.98% for Holeta branches) remain significantly higher than agreement (14.63% and 22.47%), which suggests major concerns regarding service performance, especially in waiting times and staff levels.

**Table 2:** Results of the customer feedback survey.

Customer feedback										
*List of questions*	Goro Qeransa branch					Holeta branch				
	Strongly disagree	Dis agree	Neutral	Agree	Strongly agree	Strongly disagree	Dis agree	Neutral	Agree	Strongly agree
Section I question related to resource allocation by organization										
Average	33.44	39.3	13.73	11.71	1.86	21.12	29.6	24.98	17.6	6.71
Section II question related to queue length										
Average	35.33	36.1	18.53	6.27	3.73	21.18	33.7	25.29	14.3	5.49
Section III question related to average wait times										
Average	28.67	34.7	23.73	8.8	4.13	17.57	28	32.62	14.6	7.16
Section IV question related to staff level										
Average	12	29.2	36.8	15.07	6.93	19.66	33	23.34	18.3	5.72
Total	27.36	34.8	23.2	10.46	4.17	19.88	31.1	26.56	16.2	6.27
average										

### 3.4. Waiting Time and Staff Analysis

This study analyzed the average processing time for banking services, including fund transfers, cash collections, and payments, which averaged 3.55 min based on secondary data, to evaluate operational efficiency. The M/M/c queuing model was applied to data from the Goro Qeransa and Holeta branches to assess customer service efficiency, queue dynamics, and overall system performance. The model assumes that customer arrivals follow a Poisson process, service times are exponentially distributed, servers operate independently, and customers are served on a first‐come, first‐served basis. The system is assumed to have unlimited queue capacity, ensuring that no customers are turned away.

Key parameters of the M/M/c model include the arrival rate (*λ*), the service rate (*μ*), and the number of servers (*c*), which together define the capacity and performance of the service system. The utilization factor (*ρ*) represents the proportion of system resources being actively used, whereas Little′s Law allows for the estimation of the average number of customers in the system (L) and in the queue (Lq), as well as the average time customers spend in the system (W) and waiting before service (Wq). Collectively, these metrics provide a detailed understanding of how variations in arrival rates, service rates, and server numbers influence waiting times, queue lengths, and operational efficiency. Applying this model enables identification of bottlenecks, optimal staffing levels, and resource allocation strategies for improved service delivery [40].

By implementing the M/M/c framework at the Goro Qeransa and Holeta branches, the study evaluated critical performance metrics and provided actionable insights for optimizing staff, minimizing customer waiting times, and enhancing overall operational efficiency in banking service systems. The organized results for each metric are shown in Table 3, highlighting key performance indicators that inform operational improvements.

**Table 3:** Results for each metric.

No.	Goro Qeransa branch	Customers/min (**λ**)	**p**	**L**	**L**_**q**_	**W** (minutes)	**W**_**q**_
Average		1.65	0.52	31.33	29.93	19.04	15.49

No.	Holeta branch	Customers/min (*λ*)	*p*	*L*	*L*_*q*_	*W* (minutes)	*W*_*q*_
Average		1.73	0.65	35.77	33.82	20.64	17.09

#### 3.4.1. Branch Staffing and Operational Structures

Branch banking is the primary distribution channel for the Cooperative Bank of Oromia, playing a crucial role in executing the bank′s operational strategy effectively. Table 4 outlines the staff allocation at both the Goro Qeransa and Holeta branches, revealing a structured hierarchy that includes one branch manager, two internal controllers, one manager of operations management, and various customer service officers (CSOs) such as checkers, makers, and cash‐aid officers at Goro Qeransa. Similarly, the Holeta branch features one branch manager, two internal controllers, and a diverse customer service team. This data provides valuable insights into the roles and responsibilities of staff members, which are essential for delivering high‐quality customer service.

**Table 4:** Staff allocation for both branches.

Staff allocation			
Goro Qeransa branch	No. of staff on position	Holeta branch	No. of staff on position
	12		19

#### 3.4.2. Customer Flow Patterns and Trends Analysis

**Figure 4:**
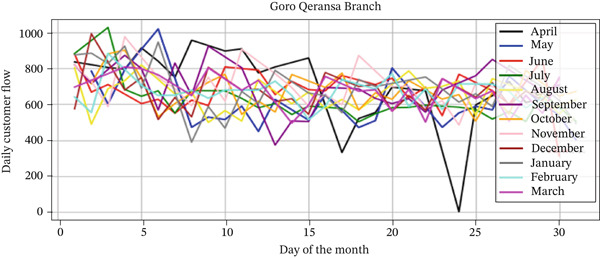
Daily customer flow by month (Goro Qeransa branch).

**Figure 5:**
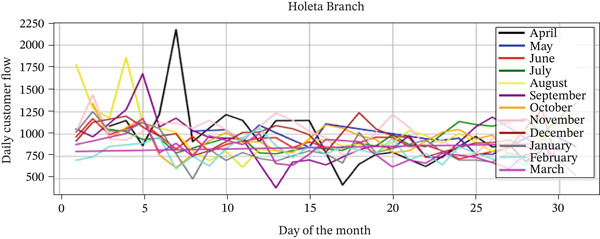
Daily customer flow by month (Holeta branch).

The analysis of customer flow data from the Goro Qeransa and Holeta branches, covering the period from April 1, 2023, to March 30, 2024, reveals valuable insights into customer visitation patterns and interactions. The line charts illustrate the daily customer flow over the year, highlighting fluctuations in visitation at both branches (Figures 4 and 5). Table 5 shows the average monthly customer flow at Goro Qeransa and Holeta branches from April 2023 to March 2024, revealing that Goro Qeransa had an average of 664.51 daily customers and Holeta had 923.60 daily customers, which helps in understanding trends and optimizing resource allocation and service strategies for both branches.

**Table 5:** Monthly average customer flow.

Daily customer flow average for each month		
For 12 months	Goro Qeransa branch	Holeta branch
Total average	664.51	923.6

#### 3.4.3. Evaluating Staffing Levels′ Impact on Waiting Time

This analysis of customer flow at the Goro Qeransa and Holeta branches explores the relationship between server counts, waiting times, and customer numbers using regression and correlation analysis. At Goro Qeransa, an average of 664.51 customers results in a 19.04 min waiting time with three servers, whereas Holeta, with 923.60 customers, has a 20.64 min waiting time with four servers. Both branches show a negative correlation between staffing levels and waiting times, highlighting the importance of adequate staffing for improved service efficiency.

The regression variables in (Equations (1), (2), (3) and (4)) have been clearly defined: **y** represents the dependent variable, either waiting time or the average number of customers in the system, whereas **x** represents the independent variable, the number of servers. The regression parameters were estimated using least‐squares linear regression based on operational data collected at the Goro Qeransa and Holeta branches, with correlation coefficients confirming that increasing staffing levels reduce waiting times and customer load.

• Goro Qeransa

Regression equation (linear) for waiting time:

(1) y=−1.410013.3315∗x+

Regression equation (linear) for average customers in system:

(2) y=−1.951818.5215∗x+

Correlation coefficient between number of servers and waiting time:

r=−0.8086

• Holeta

Regression equation (linear) for waiting time:

(3) y=−0.851111.3746∗x+ 

Regression equation (linear) for average customers in system:

(4) y=−1.637521.9611∗x+

Correlation coefficient between number of servers and waiting time:

r=−0.7500

This analysis of the Goro Qeransa and Holeta branches shows that increased server counts reduce customer waiting times, as demonstrated by regression and correlation analysis. Goro Qeransa averages 664.51 customers with a 19.04 min waiting time using three servers, whereas Holeta averages 923.60 customers with a 20.64 minute waiting time using four servers. Both branches reveal strong negative correlations between server availability and waiting times, highlighting the importance of adequate staffing for efficient service. The regression equations for waiting times indicate that increasing the number of servers decreases waiting times, with correlation coefficients of −0.8086 for Goro Qeransa and −0.7500 for Holeta, further demonstrating the impact of server availability on customer service performance.

#### 3.4.4. Data Integration and Optimization Analysis

Optimizing staff levels is critical for improving service efficiency and customer satisfaction at the Goro Qeransa and Holeta branches. In this study, a combination of LSTM neural network, regression analysis, and the M/M/c queue model was employed to support data‐driven staffing decisions. LSTM models were used to forecast customer flow, whereas regression analysis quantified the relationship between staffing levels and waiting times. An LSTM network is used to forecast customer flow at both branches, capturing long‐term dependencies in sequential data. The architecture includes an input layer matching the historical sequence length, a stacked LSTM with 50 neurons in the first layer (tanh, return_sequences = true) and 25 neurons in the second layer (tanh), followed by a dense output layer with one neuron and linear activation. The model is trained with the Adam optimizer, MSE loss, 100 epochs, and a batch size of 8, allowing it to learn nonlinear and complex temporal patterns more effectively than traditional ARIMA or exponential regression models. The M/M/c queue model evaluated queuing dynamics to determine the optimal number of servers required to meet target service levels. By integrating these techniques, the study was able to predict customer patterns accurately, minimize waiting times, and ensure efficient allocation of service resources. The process utilizes the Anaconda Jupyter library for running and coding. It starts with importing necessary libraries, such as pandas and numpy, and loading monthly average customer flow data into a DataFrame. Subsequently, the data are normalized and sequences are created for LSTM model training. After building and training the LSTM model, regression analysis is performed using scikit‐learn. Finally, Matplotlib is utilized for visualization, with the entire workflow being executed and coded within the Anaconda Jupyter environment as the next output for both branches. The optimization process involved systematic preparation and analysis of operational data. Initially, the data were collected, cleaned, and normalized to ensure compatibility with the modeling framework.

Time‐series sequences were then generated for LSTM input, and the data were partitioned into training and testing sets based on 80% of the dataset for model training and 20% of the dataset for testing the model. The LSTM model was constructed and trained using historical customer flow data, and its performance was evaluated using MSE and *R* ^2^ metrics. Forecasted customer arrivals were then combined with regression analysis to estimate the relationship between staffing levels and waiting times, followed by prediction of the optimal number of servers needed to achieve the target waiting time. Finally, the results were visualized to facilitate managerial decision‐making.

The results demonstrated high predictive accuracy and provided clear guidance for staffing optimization. At the Goro Qeransa branch, the MSE for waiting time was 0.0491, with *R* ^2^ scores of 88.17% for training and 99.64% for testing, whereas the MSE for total customers was 0.0985, with corresponding *R* ^2^ scores of 87.94% and 99.63%. The optimal number of servers to maintain a target waiting time of 3.55 min was six (6), resulting in an average of approximately five customers in the system. At the Holeta branch, the MSE for waiting time was 0.0577, with *R* ^2^ scores of 88.17% for training and 99.64% for testing, and the MSE for total customers was 0.2210, with *R* ^2^ scores of 88.00% and 99.63%. The optimal staffing level was seven (7) servers, corresponding to an average of approximately seven customers in the system.

**Figure 6:**
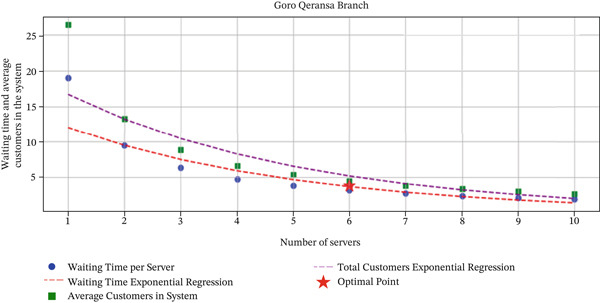
Optimized servers versus waiting time and average customers at Goro Qeransa.

**Figure 7:**
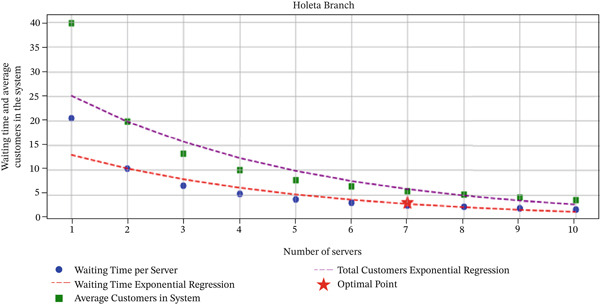
Optimized servers versus waiting time and average customers at Holeta.

These findings, illustrated in Figures 6 and 7, indicate that the integration of predictive analytic and queuing models provides actionable insights for optimizing staffing levels in banking operations. By leveraging this methodology, branch managers can enhance operational efficiency, maintain target‐waiting times, and improve overall customer satisfaction, demonstrating the practical value of combining advanced forecasting techniques with classical queuing theory.

### 3.5. Summary and Output Analysis

This section offers an analysis of the performance and sentiment data from the Goro Qeransa and Holeta branches of the Cooperative Bank of Oromia.

#### 3.5.1. Assessment Discussion

This section analyzes staff and customer feedback, along with queue model metrics from the Goro Qeransa and Holeta branches, highlighting operational performance issues. At the Goro Qeransa branch, staff feedback indicates a neutral to agreeable sentiment among employees, whereas customer feedback shows significant dissatisfaction. Key metrics reveal an average of 1.65 customers per minute and considerable waiting times, reflecting challenges in managing customer flow, with an average daily traffic of 664.51 customers supported by 12 staff members. In addition, the Holeta branch exhibits a less favorable staff sentiment, with customer feedback also indicating dissatisfaction, though slightly less pronounced than at Goro Qeransa. The queue metrics are higher, with 1.73 customers per minute and longer wait times, suggesting greater congestion and operational inefficiencies. The Holeta branch manages an average of 923.60 customers daily, supported by 19 staff members. Overall, both branches require improvements in employee satisfaction and customer service, as evidenced by the disconnect between staff perceptions and customer experiences. The significant waiting times and congestion highlight the necessity for advanced queue management and optimized staffing during peak hours. Addressing these feedback insights is essential for enhancing service quality and operational efficiency, ultimately fostering better relationships between staff and customers and increasing overall satisfaction.

#### 3.5.2. Discussion on Optimization Results

The findings of this study confirm that queuing theory effectively enhances service efficiency and reduces waiting times in banking systems. Using the M/M/c queue model to determine optimal service counters aligns with recent research showing that queue‐based models can improve operational performance and minimize customer wait times [41]. The study′s assumptions of Poisson customer arrivals and exponential service times align with standard queuing models commonly used in banking and service industries to estimate performance measures such as waiting time, queue length, and server utilization [42].

In addition, this research extends previous work by integrating the long short‐term memory neural network and regression analysis with classical queuing models. Recent studies have shown that LSTM networks are effective for modeling time‐series patterns and predicting complex sequential data, making them suitable for forecasting demand and customer activity in financial systems [43, 44]. The integration of predictive machine learning techniques with queuing theory provides a more proactive framework for service optimization.

Overall, results showed that six servers at Goro Qeransa and seven at Holeta could maintain an average waiting time of 3.55 min, demonstrating that predictive and queuing models can effectively improve operational efficiency and customer satisfaction. The outcome confirms that combining predictive analytics and queuing‐based models can improve staffing decisions and resource allocation in banking operations. This approach enables financial institutions to anticipate fluctuations in customer demand and adjust service capacity accordingly, leading to improved service quality and customer satisfaction.

## 4. Conclusion

The study evaluated staffing levels and customer service efficiency at the Goro Qeransa and Holeta branches of the Cooperative Bank of Oromia. The integration of LSTM neural network, regression analysis, and the M/M/c queuing model enabled the prediction of customer flow, the identification of service bottlenecks, and the determination of the optimal number of servers. The results indicate that staffing six servers at Goro Qeransa and seven at Holeta can maintain an average waiting time of approximately 3.55 min, which improves operational efficiency while handling daily customer volumes of 664.5 and 923.6, respectively. Despite increased staffing, the Holeta branch exhibited higher congestion, suggesting that variability in arrival rates and differences in service times must be considered in branch‐specific optimization. These findings support the use of predictive and queuing‐based models for resource allocation and provide actionable insights for improving service delivery and customer satisfaction in banking operations. The future direction is integrating additional customer behavior data and exploring hybrid queuing‐forecasting models for broader applicability in the banking sector.

## Author Contributions

**Tadele Mamo:** conceptualization, data curation, methodology, visualization, supervision. **Dame Tolosa:** formal analysis, investigation, project administration. **S. Nagarajan:** original draft, software, formal analysis, methodology. Velmurugan Paramasivam: validation, writing – review and editing. **Wosen Haile Mariam Asfaw:** data analysis and investigation. S. Umamaheswaran: software, data analysis, formal analysis.

## Funding

No funding was received for this manuscript.

## Conflicts of Interest

The authors declare no conflicts of interest.

## Data Availability

All necessary data are included in the paper.

## References

[1] Werner R. , A Lost Century in Economics: Three Theories of Banking and the Conclusive Evidence, International Review of Financial Analysis. (2016) 46, 361–379, 10.1016/j.irfa.2015.08.014, 2-s2.0-84949678683.

[2] Challoumis C. and Eriotis N. , Evolution of Banking Systems: A Comprehensive Historical Analysis, Journal of Contemporary Research in Business, Economics and Finance. (2025) 7, no. 1, 1–21, 10.55214/jcrbef.v7i1.4245.

[3] Llewellyn D. T. , Edey M. , Banking in the 21st Century: The Transformation of an Industry, The Future of the Financial System, 1996, Reserve Bank of Australia, 142–192.

[4] Dinçkol D. , Ozcan P. , and Zachariadis M. , Regulatory Standards and Consequences for Industry Architecture: The Case of UK Open Banking, Research Policy. (2023) 52, no. 6, 104760, 10.1016/j.respol.2023.104760.

[5] Chakravorti S. , Customer Relationship Management: A Global Approach, 2023, SAGE Publications Ltd.

[6] Momparler A. , Lassala C. , and Ribeiro D. , Efficiency in banking Services: A Comparative Analysis of Internet-Primary and Branching Banks in the US, Service Business. (2013) 7, no. 4, 641–663, 10.1007/s11628-012-0179-1, 2-s2.0-84887401116.

[7] National Bank of Ethiopia , Annual Report on Regulatory Developments in Ethiopian Banking, 2021, NBE Publications.

[8] Hillier F. S. and Lieberman G. J. , Introduction to Operations Research, 2001, 7th edition, McGraw-Hill.

[9] Kleinrock L. , Queueing Systems: Theory and Applications, 1976, Wiley.

[10] Najaf A. and Fakhri A. , Use the Queue Theory to Assess the Quality Standards of Service Systems to Achieve the Customer′s Desire, World Economics and Finance Bulletin. (2024) 36, 117–126, 10.13140/RG.2.2.13942.20807.

[11] Mansour A. , Rowlands H. , Al-Gasawneh J. A. , Nusairat N. M. , Al-Qudah S. , Shrouf H. , and Akhorshaideh A. H. , Perceived Benefits of Training, Individual Readiness for Change, and Affective Organizational Commitment Among Employees of National Jordanian Banks, Cogent Business & Management. (2022) 9, no. 1, 1966866, 10.1080/23311975.2021.1966866.

[12] Larson R. C. and Pinker E. J. , Melnick E. L. , Nayyar P. R. , Pinedo M. L. , and Seshadri S. , Staffing Challenges in Financial Services, Creating Value in Financial Services, 2000, Springer, 327–356, 10.1007/978-1-4615-4605-4_17.

[13] Yas H. , Mardani A. , and Alfarttoosi A. , The Major Issues Facing Staff in Islamic Banking Industry and Its Impact on Productivity, Contemporary Economics. (2020) 14, no. 3, 392–405, 10.5709/ce.1897-9254.412.

[14] Al-Suraihi W. A. , Samikon S. A. , Al-Suraihi A. H. A. , and Ibrahim I. , Employee Turnover: Causes, Importance and Retention Strategies, European Journal of Business Management and Research. (2021) 6, no. 3, 1–10, 10.24018/ejbmr.2021.6.3.893.

[15] Bassey J. U. , Boniface O. P. , Abiji A. E. , and Chidubem B. S. , Effects of Waiting Line Management on Customer Satisfaction: A Study of Selected Banks in Ogoja, Nigeria, International Journal of Economics and Business Management. (2025) 1, no. 2, 279–296, 10.59568/IJEBM-2025-1-2-20.

[16] Shifa Fathima J. , Challenge Management of Banking Services–With Special Reference to Virtual Banking Service Challenges, Shanlax International Journal of Management. (2020) 7, no. 3, 57–66, 10.34293/management.v7i3.1620.

[17] Zemene D. and Hiluf B. , The Influence of Waiting Lines Management on Customer Satisfaction in Commercial Bank of Ethiopia, Financial Markets, Institutions and Risks. (2019) 3, no. 3, 5–12, 10.21272/fmir.3(3).5-12.2019.

[18] Tan L. H. , Chew B. C. , and Hamid S. R. , Relationship Between Service Quality and Customer Satisfaction: A Study of Malaysian Banking Industry, International Journal of Productivity and Quality Management. (2016) 19, no. 1, 38–50, 10.1504/IJPQM.2016.078008, 2-s2.0-84982793386.

[19] Dunfa B. A. , Service Quality and Customer Satisfaction (The Case of Cooperative Bank of Oromia, Addis Abeba, Ethiopia), Research Journal of Finance and Accounting. (2020) 11, no. 17, 10–22, 10.7176/RJFA/11-17-02.

[20] Rece L. , Vlase S. , Ciuiu D. , Neculoiu G. , Mocanu S. , and Modrea A. , Queueing Theory-Based Mathematical Models Applied to Enterprise Organization and Industrial Production Optimization, Mathematics. (2022) 10, no. 14, 10.3390/math10142520.

[21] Kalwar M. , Marri H. , Khan M. , and Khaskheli S. , Applications of Queuing Theory and Discrete Event Simulation in Health Care Units of Pakistan, International Journal of Science and Engineering Investigations. (2021) 10, no. 109, 6–18.

[22] Gumus S. , Bubou G. , and Oladeinde M. , Application of Queuing Theory to a Fast Food Outfit: A Study of Blue Meadows Restaurant, Independent Journal of Management & Production. (2017) 8, no. 2, 441–458, 10.14807/ijmp.v8i2.576.

[23] Yifter T. , Mengstenew M. , Yoseph S. , and Moges W. , Modeling and Simulation of Queuing System to Improve Service Quality at Commercial Bank of Ethiopia, Cogent Engineering. (2023) 10, no. 2, 2274522, 10.1080/23311916.2023.2274522.

[24] Jenčová E. , Koščák P. , and Koščáková M. , Dimensioning the Optimal Number of Parallel Service Desks in the Passenger Handling Process at Airports Considered as a Queueing System—Case Study, Aerospace. (2023) 10, no. 1, 10.3390/aerospace10010050.

[25] Shortle J. F. , Thompson J. M. , Gross D. , and Harris C. M. , Fundamentals of Queueing Theory, 2018, 5th edition, Wiley, 10.1002/9781119453765, 2-s2.0-85050743078.

[26] Siami-Namini S. , Tavakoli N. , and Namin A. S. , A Comparative Analysis of Forecasting Financial Time Series Using ARIMA, LSTM, and BiLSTM, 2019, arXiv preprint arXiv:1911.09512 10.48550/arXiv.1911.09512.

[27] Kader N. I. A. , Yusof U. K. , Khalid M. N. A. , Husain N. R. N. , and Al-Sharafi M. A. , Al-Emran M. , and Al-Kabi M. N. , Shaalan K. , A Review of Long Short-Term Memory Approach for Time Series Analysis and Forecasting, Proceedings of the 2nd International Conference on Emerging Technologies and Intelligent Systems (ICETIS 2023), 2023, Springer, 10.1007/978-3-031-20429-6_2.

[28] Palihakkara K. and Jayakody M. , A Comparative Study on Deep Learning Models for Time-Series Forecasting, 2026, 10.20944/preprints202601.1962.v1.

[29] Arifin S. , Wijaya A. K. , Nariswari R. , Yudistira I. G. A. A. , Suwarno , Faisal , and Wihardini D. , Long Short-Term Memory (LSTM): Trends and Future Research Potential, International Journal of Emerging Technology and Advanced Engineering. (2023) 13, no. 5, 24–34, 10.46338/ijetae0523_04.

[30] Hamdan A. M. , Al-Salaymeh A. , AlHamad I. M. , Ikemba S. , and Ewim D. R. E. , Predicting Future Global Temperature and Greenhouse Gas Emissions via LSTM Model, Sustainable Energy Research. (2023) 10, 10.1186/s40807-023-00092-x.

[31] Bareth R. , Yadav A. , Gupta S. , and Pazoki M. , Daily Average Load Demand Forecasting Using LSTM Model Based on Historical Load Trends, IET Generation, Transmission & Distribution. (2024) 18, no. 5, 952–962, 10.1049/gtd2.13132.

[32] Lu J. , Guo Y. , Wang M. , Luo Y. , Zeng X. , Miao X. , Zaman A. , Yang H. , Cao A. , and Kang Y. , Determining Acute Ischemic Stroke Onset Time Using Machine Learning and Radiomics Features of Infarct Lesions and Whole Brain, Mathematical Biosciences and Engineering. (2024) 21, no. 1, 34–48, 10.3934/mbe.2024002, 38303412. 38303412

[33] Qin X. , Leng C. , and Dong X. , A Hybrid Ensemble Forecasting Model of Passenger Flow Based on Improved Variational Mode Decomposition and Boosting, Mathematical Biosciences and Engineering. (2023) 21, no. 1, 300–324, 10.3934/mbe.2024014, 38303424. 38303424

[34] Zhou H. , Ai Q. , and Li R. , Short-Term Multi-Energy Load Forecasting Method Based on Transformer Spatio-Temporal Graph Neural Network, Energies. (2025) 18, no. 17, 10.3390/en18174466.

[35] Tian C. , Lin L. , Yan Y. , Wang R. , Wang F. , and Chi Q. , Photovoltaic Power Prediction Based on Dilated Causal Convolutional Network and stacked LSTM, Mathematical Biosciences and Engineering. (2023) 21, no. 1, 1167–1185, 10.3934/mbe.2024049, 38303459. 38303459

[36] Creswell J. W. and Creswell J. D. , Research Design: Qualitative, Quantitative, and Mixed Methods Approaches, 2022, 6th edition, SAGE Publications.

[37] Israel G. D. and Tounsi G. , Determining Sample Size (PEOD6), 2025, University of Florida, IFAS Extension.

[38] Adeniran A. , Understanding Cronbach′s Alpha in Social and Management Studies, Current Science Research Bulletin. (2025) 2, no. 2, 11–16.

[39] Khan M. , Optimizing Web Surveys in Research: Methodological Considerations and Validity Aspects, International Journal of Research and Scientific Innovation. (2024) 11, no. 4, 75–105, 10.51244/IJRSI.2024.1104007.

[40] Yakubu A.-W. N. and Najim U. , An Application of Queuing Theory to ATM Service Optimization: A Case Study, Mathematical Theory and Modeling. (2014) 4, no. 6, 11–24.

[41] Abushaala M. and Essdai A. , Applications of Waiting Lines Models for Improving the Performance Levels of Banking Service System, Journal of Technology Research. (2024) 2, no. 3, 13–23, 10.26629/jtr.2024.03.

[42] Sherzer E. , Baron O. , Krass D. , and Resheff Y. , ApproximatingG(t)/GI/1queues With Deep Learning, European Journal of Operational Research. (2025) 322, no. 3, 889–907, 10.1016/j.ejor.2024.12.030.

[43] Yang Z. , Zhang Y. , and Yu L. , Predicting Bank Users′ Time Deposits Based on LSTM-Stacked Modeling Learning, Applied Theory and Artificial Intelligence in Machine Learning. (2024) 3, no. 3, 172–182, 10.56578/ataiml030304.

[44] Kilinc M. , LSTM-Based Time Series Forecasting of User-Derived Quality Signals in Mobile Banking Systems, Systems. (2025) 13, no. 11, 10.3390/systems13110949.

